# Characteristics and performance of a bispecific F (ab'gamma)2 antibody for delivering saporin to a CD7+ human acute T-cell leukaemia cell line.

**DOI:** 10.1038/bjc.1991.291

**Published:** 1991-08

**Authors:** D. J. Flavell, S. Cooper, B. Morland, S. U. Flavell

**Affiliations:** University Department of Pathology, Southampton General Hospital, UK.

## Abstract

We have investigated the efficacy of a F(ab'gamma)2 bispecific antibody (BsAb) with dual specificity for the CD7 molecule in one Fab arm and for the ribosome inactivating protein (rip) saporin in the other arm, for delivering saporin to the acute T-cell leukaemia cell line HSB-2. Saporin titration experiments revealed that BsAb increased the toxicity of saporin 435-fold for HSB-2 cells, reducing the IC50 for saporin alone from 0.1 mumol to 0.23 nmol when BsAb was included. The rate of protein synthesis inactivation brought about by BsAb-mediated toxin delivery to HSB-2 cells was very similar to that described for conventional immunotoxins (IT's) with a t10 (time taken for a one log inhibition of protein synthesis compared with controls) of 46 h obtained at a saporin concentration of 1 nmol and 226 h at 0.1 nmol. BsAb titration studies demonstrated a clear dose response effect of BsAb concentration on target cell protein synthesis inhibition and cell proliferation. The absolute specificity of toxin delivery was unequivocally demonstrated by a failure of BsAb to deliver an effective dose of saporin to the CD7- cell line HL60 and by the blocking of BsAb-mediated delivery of saporin to HSB-2 cells with an excess of F(ab)2 fragments of the anti-CD7 antibody, HB2. These studies have clearly demonstrated the effectiveness of this BsAb for delivering saporin to a T-ALL cell line utilising CD7 as the target molecule on the cell surface. BsAb's would therefore appear to offer a realistic alternative to IT's for toxin delivery to tumour cells and may even offer certain advantages over conventional IT's for clinical use.


Br. J. Cancer (1991), 64, 274-280
© Macmillan Press Ltd., 1991

### Characteristics and performance of a bispecific F $(ab'\gamma)_2$ antibody for delivering saporin to a $CD7^+$ human acute T-cell leukaemia cell line

D.J. Flavell[1], S. Cooper[1], B. Morland[2] & S.U. Flavell[1]

[1]*Monoclonal Antibody Unit, University Department of Pathology and* [2]*University Department of Child Health, Southampton General Hospital, Southampton SO9 4XY, UK.*

**Summary** We have investigated the efficacy of a $F(ab'\gamma)_2$ bispecific antibody (BsAb) with dual specificity for the CD7 molecule in one Fab arm and for the ribosome inactivating protein (rip) saporin in the other arm, for delivering saporin to the acute T-cell leukaemia cell line HSB-2. Saporin titration experiments revealed that BsAb increased the toxicity of saporin 435-fold for HSB-2 cells, reducing the $IC_{50}$ for saporin alone from 0.1 μmol to 0.23 nmol when BsAb was included. The rate of protein synthesis inactivation brought about by BsAb-mediated toxin delivery to HSB-2 cells was very similar to that described for conventional immunotoxins (IT's) with a $t_{10}$ (time taken for a one log inhibition of protein synthesis compared with controls) of 46 h obtained at a saporin concentration of 1 nmol and 226 h at 0.1 nmol. BsAb titration studies demonstrated a clear dose response effect of BsAb concentration on target cell protein synthesis inhibition and cell proliferation. The absolute specificity of toxin delivery was unequivocally demonstrated by a failure of BsAb to deliver an effective dose of saporin to the $CD7^-$ cell line HL60 and by the blocking of BsAb-mediated delivery of saporin to HSB-2 cells with an excess of $F(ab)_2$ fragments of the anti-CD7 antibody, HB2. These studies have clearly demonstrated the effectiveness of this BsAb for delivering saporin to a T-ALL cell line utilising CD7 as the target molecule on the cell surface. BsAb's would therefore appear to offer a realistic alternative to IT's for toxin delivery to tumour cells and may even offer certain advantages over conventional IT's for clinical use.

Despite the great advances that have been made in the treatment of both adult and childhood acute lymphoblastic leukaemia, there are still approximately one half of all adults and one third of all children who relapse during or following the cessation of treatment (Chessells, 1987). The prognosis for these patients who continue with further conventional chemotherapy following relapse is poor (Champlin & Gale, 1989). The main current therapeutic strategy for patients with relapsed acute leukaemia that have failed primary treatment, is very high dose ablative chemo-radiotherapy followed by allogeneic or autologous bone marrow transplantation (Report from the Working Party on Leukaemia, 1988). Allogeneic transplantation offers a considerably higher success rate in terms of cure than autografting but HLA matched donors are available to only a small minority in need of a transplant (Kersey *et al.*, 1987). The challenge therefore is to devise alternative therapies which will prove effective for those patients who do relapse and which might also prove beneficial as a compliment to conventional treatment, improving the overall cure rate and reducing the toxicity of treatment overall. Targeted therapy utilising monoclonal antibodies for selectivity delivering drugs, toxins or radioisotopes to target tumour cells offers one possible alternative method for the elimination of residual neoplastic cells.

Immunotoxins (IT) are hybrid molecules comprised of a toxin species covalently coupled, usually through a cleavable thiol bond to an antibody (Hertler & Frankel, 1989). The antibody confers target cell specificity on the toxin and provides the means for entry of the toxin into the cell following internalisation of the IT by endocytotis (Press *et al.*, 1988). The subsequent cleavage of the toxin species from the antibody and its route of translocation from the endosome compartment to target ribosomes in the cytosol all act to influence the efficacy of any particular IT. IT's have proven highly effective at selectively killing a variety of target tumours cells *in vitro* (Bregni *et al.*, 1989; Ghetie *et al.*, 1988; Strong *et al.*, 1985) and *in vivo* (Blythman *et al.*, 1986; Thorpe *et al.*, 1985; FitzGerald *et al.*, 1986)) and clinical trials of IT's for a variety of malignant neoplasias are now underway (Spitler, 1988; Laurent *et al.*, 1988; Byers *et al.*, 1990). A number of major obstacles need to be overcome before IT-type treatments make any major impact in the clinical treatment of cancer. Problems exist regarding the slow rate with which IT's kill target cells (Casellas *et al.*, 1984) and linked to this is the short half life of IT conjugates *in vivo* (Scott *et al.*, 1987), though with regard to the latter recent improvements in coupling chemistry has led to a prolongation of *in vivo* half life of IT's constructed with Ricin A chain (Thorpe *et al.*, 1988). Fortuitously, IT's constructed with saporin have an apparent longer half life *in vivo* in the mouse (Blakey *et al.*, 1988), though saporin IT's appear to be up to 30 times more toxic than ricin A chain IT's for liver parenchyma. Strategies need to be developed to overcome these and similar types of problem which will be arrived at only by careful and meticulous study of both the *in vitro* and *in vivo* properties and characteristics of IT's or BsAb's.

As an alternative to conventional IT's some workers have constructed bispecific antibodies with dual specificities for a tumour associated target molecule in one of the Fab arms and for a toxin in the other (Webb *et al.*, 1985; Glennie *et al.*, 1988). The major advantage here is that chemical procedures for coupling the toxin to the antibody are no longer required and that the BsAb may be administered and allowed to localise to the tumour cell surface *prior* to administration of toxin, a strategy that may serve to reduce the immunogenicity and perhaps also the toxicity of the complex. We describe here the characteristics and performance of the BsAb HB2 × DB7-18 for delivering the ribosome inactivating protein (rip) saporin to the T-ALL cell line HSB-2 via the CD7 cell surface molecule. We demonstrate that this BsAb is probably just as effective as an IT for selectively delivering a cytotoxic dose of saporin to this T-ALL cell line.

### Materials and methods

### *Human acute leukaemia cell lines*

Three established human acute leukaemia cell lines were used for the purposes of this study, the $CD7^+$ T-ALL cell line HSB-2, the $CD7^-$ promyelocytic leukaemia cell line HL60 and the CD7 weakly positive T-ALL cell line HPB-ALL. All three cell lines were maintained in the logarithmic phase of growth in RPMI 1640 medium supplemented with 10% foetal calf serum (Gibco), 1 mmol glutamine, 1 mmol sodium pyruvate, 100 IU $ml^{-1}$ benzyl penicillin and 100 μg $ml^{-1}$

Correspondence: D.J. Flavell
Received 21 January 1991; and in revised form 12 March 1991.

streptomycin sulphate. Cells were only used when the cell viability of the cultures exceeded 95%.

*Saporin*

The ribosome inactivating protein (*rip*) Saporin was purified from the seeds of *Saponaria officinalis* as previously described (Stirpe *et al.*, 1983). SDS-polyacrylamide gel electrophoresis of the final preparation revealed a single band with an $M_r$ corresponding to 29,500 daltons. The concentration of saporin was estimated from its absorbance at 280 nmol taking $A_{1cm.280}$ as equal to 0.6.

*Monoclonal antibodies*

The anti-saporin MoAb DB7-18 (Glennie *et al.*, 1987) and the anti-CD7 MoAb HB2 were used in this study. Hybridoma cells secreting each of these antibodies were injected into pristane primed mice for the production of antibody containing ascitic fluids. The 7S IgG fractions of ascitic fluid were isolated by precipitation with 2 M ammonium sulphate followed by ion exchange chromatography on Trisacryl-M-DEAE. $F(ab')_2$ fragments for each MoAb were prepared by limited proteolysis with pepsin at pH 4.2 as described previously (Glennie *et al.*, 1988).

*Construction of $F(ab'\gamma)_2$ bispecific antibody*

A Heterodimeric $F(ab'\gamma)_2$ bispecific antibody (BsAb) containing one Fab' arm from HB2 and the other Fab' arm from DB7-18 (HB2 × DB7-18) was constructed as described by Glennie *et al.* (1987). Briefly, $F(ab')_2$ molecules from each antibody were reduced to obtain Fab' fragments with hinge region SH groups, Fab' (SH). The SH groups on one of the Fab' (SH) species were then fully alkylated with excess 0-phenylenedimaleimide to provide free maleimide groups. The two preparations, Fab' (mal) and Fab' (SH) were then combined under conditions which allowed cross linking of the maleimide and the SH groups and avoided reoxidation of the SH groups. The final products were reduced and alkylated to remove any minor untoward products which may have formed by oxidation or disulphide exchange and the final mixture fractionated according to size by chromatography on Ultragel AcA44 (LKB-Produkter AB, Bromma, Sweden).

*Analysis of cell surface expression of CD7 by flow cytometry*

Cells were anlaysed for surface expression of CD7 by flow cytometry. Cells were incubated for 1 h at room temperature with a saturating concentration of HB2 antibody. Following the first incubation cells were washed twice and incubated with a 1:20 dilution of fluorescein isothiocyanate conjugated $F(ab')_2$ fragments of a rabbit anti-mouse immunoglobulins antisera (Sigma Chemical Co., Poole, Dorset, UK). Cell surface expression of CD7 was then measured by flow cytometry.

*$^3$H-leucine incorporation by HSB-2 cells*

Protein synthesis levels in HSB-2 cells exposed to BsAb and saporin were measured by $^3$H-leucine incorporation into cellular proteins. The method used was essentially that described previously. Briefly triplicate cultures of HSB-2 cells at a density of $1 \times 10^5$ cells/well in 96 well microculture plates were exposed for 48 h at 37°C to BsAb and saporin at each experimental concentration. Cells were then pulsed for 12 h with 1.0 μCi $^3$H-leucine (TRK 510, Amersham International, Amersham, UK) and finally harvested onto glassfibre filters using a Skatron cell harvester. The amount of radioactive leucine incorporated by cells was measured by scintillation counting the harvested cells on the glassfibre discs in a Packard scintillation counter. Results obtained for experimental cultures are expressed as a percentage of the amount of $^3$H-leucine incorporation observed in untreated conrol cultures.

*Kinetic studies*

The kinetics of protein synthesis inactivation by BsAb and saporin was determined in 96 well microcultures of HSB-2 cells exposed to a various concentrations of saporin (range $10^{-11}$ to $10^{-7}$ M) together with BsAb at 0.1 $\mu g\,ml^{-1}$. HSB-2 cells were incubated for 2 h in supplemented leucine-free RPMI medium at 37°C and then triplicate samples of $1 \times 10^5$ cells added to wells of a 96 well microculture plate containing each appropriate concentration of saporin and BsAb in supplemented leucine-free RPMI. Microculture plates were maintained at 37°C in a humidified atmosphere of 5% $CO_2$ in air and at 2, 6, 12, 24 and 48 h 1.0 μCi $^3$H-leucine was added to each well of the appropriate cultures and cells harvested after a 1 h pulse onto glassfibre mats as described above. Regression analysis of $^3$H-leucine incorporation levels (expressed as a percentage of control cultures) *vs* each timepoint studied was undertaken for each saporin concentration employed. The time taken to reduce the protein synthesis level of HSB-2 cells by one log is defined as the $t_{10}$ and was obtained from the intercept point of the regression line with the 10% level on the regression chart.

*Long term HSB-2 cultures*

$1 \times 10^5$ HSB-2 cells were continuously exposed to various concentrations of saporin and BsAb in supplemented RPMI medium in 25 cm$^3$ tissue culture flasks maintained at 37°C in a humidified atmosphere of 5% $CO_2$ in air. Daily viable cell counts were undertaken for a 10 day period and $^3$H-leucine incorporation estimated as described above in parallel 96 well microcultures of HSB-2 cells exposed in triplicate to the same concentrations of BsAb and saporin.

### Results

*Cell surface expression of CD7 by HSB-2, HPB-ALL and HL60 cells*

Flow cytometry revealed that 98% of HSB-2 cells expressed CD7 strongly with a mean fluorescent intensity of 259 (arbitrary units). Ninety five per cent of HPB-ALL cells were effectively negative or very weakly positive for CD7 expression but a subpopulation of $<5\%$ of the cells showed moderate expression with a mean fluorescent intensity within this population of 63 arbitrary units. All HL60 cells were effectively negative for CD7 expression with a mean fluorescent intensity of only 8.3 arbitrary units.

*Specific delivery of saporin to the CD7$^+$ cell line HSB-2 by BsAb*

The $F(ab'\gamma)_2$ BsAb HB2 × DB7-18 with anti-CD7 specificity in one Fab arm and anti-saporin specificity in the other arm was constructed in order to investigate its effectiveness at specifically delivering a lethal amount of saporin to the CD7$^+$ T-ALL cell line, HSB-2. The CD7$^-$ promyelocytic cell line HL60 and the weakly positive T-ALL cell line HPB-ALL were used as a check of target cell specificity in related experiments. All results reported here are representative of each experiment repeated on at least three separate occasions.

*Bispecific antibody and saporin titration against HSB-2 cells*

Triplicate cultures of $1 \times 10^5$ HSB-2 cells were exposed for 48 h to BsAb at 0.1 $\mu g\,ml^{-1}$ together with increasing concentrations of saporin (ranging from $10^{-12}$ to $10^{-6}$ M). Identical numbers of cells were exposed to concentrations of saporin alone or saporin + an equimolar mixture of the two different $F(ab')_2$ fragments (0.05 $\mu g\,ml^{-1}$ of each) from which the BsAb was constructed. Cultures of untreated control cells were set up in wells containing medium alone. $^3$H-leucine incorporation ws evaluated in all cell cultures after 48 h of exposure and results expressed as a percentage of the control levels are shown in Figure 1. An $IC_{50}$ of 0.1 μmol was obtained for saporin alone but when the BsAb was included the $IC_{50}$ was reduced to 0.23 nmol this representing a 435-fold increase in toxicity. $F(ab'\gamma)_2$ fragments together with saporin had a negligible effect on toxicity ($IC_{50}$ 0.08 μmol).

In a series of similar experiments HSB-2 cells were exposed to saporin at a concentration of 0.1 μg $ml^{-1}$ ($3.3 \times 10^{-9}$ M) in the presence of varied BsAb concentrations (range 0.001 μg $ml^{-1}$ to 1 μg $ml^{-1}$). Representative results for Leucine incorporation following a 48 h exposure of these cell cultures expressed as a percentage of untreated control cells are shown in Figure 2. A clear dose response curve was seen with increasing concentrations of BsAb, the $IC_{50}$ being achieved at a BsAb concentration of 0.011 μg $ml^{-1}$. BsAb alone or $F(ab'\gamma)_2$ fragments from the HB2 + DB7-18 MoAb's present in equimolar concentrations over the entire concentration range together with saporin at 0.1 μg $ml^{-1}$ had no significant effect on protein synthesis.

**Figure 1** Protein synthesis levels in HBS-2 cells exposed to various concentrations of saporin alone (■——■) ($IC_{50}$ 0.1 μmol), or in combination with 0.1 μg $ml^{-1}$ HB2 x DB7-18 BaAb (▲——▲) ($IC_{50}$ 0.23 nmol) or $F(ab')_2$ fragments of HB2 + DB7-18 (□---□) ($IC_{50}$ 0.08 μmol). Bars indicate one standard deviation.

**Figure 2** Protein synthesis levels in HSB-2 cells exposed to various concentrations of HB2 x DB7-18 BsAb in the absence (□---□) and presence (■——■) of 0.1 μg $ml^{-1}$ saporin or to HB2 + DB7-18 $F(ab)_2$ fragments in the presence of 0.1 μg $ml^{-1}$ Saporin (▲——▲). The $IC_{50}$ was achieved at a BsAb concentration of 0.011 μg $ml^{-1}$. Bars indicate one standard deviation.

*Kinetics of BsAb action*

Experiments were conducted to determine the rate at which various concentrations of saporin (range $10^{-11}$ to $10^{-7}$ M) inactivated protein synthesis in HSB-2 cells in the presence of BsAb at 0.1 μg $ml^{-1}$. Figure 3 shows the regression analysis for the rate of protein synthesis inactivation for each saporin concentration (expressed as the log percentage control $^3$H-leucine incorporation *vs* time). The rate of inactivation is clearly concentration dependent and linear in nature. Regression coefficients obtained for each saporin concentration were $r = 0.908$ ($10^{-7}$ M), 0.912 ($10^{-8}$ M), 0.983 ($10^{-9}$ M), 0.831 ($10^{-10}$ M) and 0.610 ($10^{-11}$ M). The time taken for a one log inhibition of protein synthesis relative to an equivalent number of untreated control cells is defined as the $t_{10}$ and is calculated from the intercept point of the regression line with the 10% level on the chart, and this value, plotted against each saporin concentration is shown in a Figure 3a graph. At a saporin concentration $10^{-11}$ M no observable inhibition of protein synthesis was detectable and the $t_{10}$ value is therefore infinitely long and consequently unplottable on the graph. At a saporin concentration of $10^{-10}$ M the $t_{10}$ was shown to be 226 h whilst at saporin concentrations of $10^{-9}$, $10^{-8}$ and $10^{-7}$ M the $t_{10}$ values were shown to be 46, 30 and 20 h, respectively.

**Figure 3** Kinetics of protein synthesis inactivation in HSB-2 cells exposed to BsAb (0.1 μg $ml^{-1}$) together with saporin at concentrations ranging from $10^{-11}$ M to $10^{-7}$ M. The rate slopes shown in **b** were obtained following measurement of $^3$H-leucine incorporation at 2, 6, 12, 24 and 48 h exposure of cells to BsAb in combination with saporin at the various concentrations. **a** shows the calculated $t_{10}$ values (the time taken to reduce protein synthesis levels by 90%) plotted against the relevant saporin concentration.

*Specificity of BsAb-mediated delivery of saporin to HSB-2*

We undertook two different types of experiment in order to confirm that saporin was indeed being specifically delivered by the BsAb to HSB-2 cells via the CD7 cell surface molecule. In the first experiment the $CD7^-$ promyelocytic cell line HL60 and the CD7 weakly positive T-ALL cell line HPB-ALL were exposed to concentrations of saporin (range $10^{-12}$ to $10^{-6}$ M) together with BsAb (0.1 $\mu g\,ml^{-1}$) and $^3$H-leucine incorporation evaluated after 48 h as in previous experiments. The results are shown in Figure 4 and as can be seen the BsAb failed to deliver an effective dose of saporin to the $CD7^-$ cell line HL60 with an identical $IC_{50}$ of 90 nmol obtained for both saporin alone and for saporin in combination with BsAb. HPB-ALL, the T-ALL cell line weakly positive for CD7 was more sensitive to saporin alone giving an $IC_{50}$ value of 51 nmol. In the presence of the BsAb the $IC_{50}$ was reduced to 22 nmol, this representing a two and a half-fold increase in toxicity compared with that obtained for saporin alone.

In a second type of specificity experiment we attempted to block the binding of BsAb to the CD7 target molecule on the HSB-2 cell surface with a 10-fold excess of HB2 $F(ab')_2$. As in previous titration experiments HSB-2 cells were exposed to saporin alone (ranging from $10^{-12}$ to $10^{-6}$ M) or in combination with BsAb (0.1 $\mu g\,ml^{-1}$) in the absence and presence of a 10-fold excess of HB2 $F(ab')_2$ (1 $\mu g\,ml^{-1}$) and $^3$H-leucine incorporation evaluated after a 48 h exposure. The results in Figure 5 show clearly that $F(ab')_2$ shifted the titration curve to the right, significantly reducing the cytotoxicity of the BsAb from an $IC_{50}$ of 0.22 nmol without $F(ab')_2$ to 73 nmol with $F(ab')_2$ a 332-fold decrease in toxicity demonstrating effective blocking of BsAb binding by $F(ab')_2$.

*Effects of BsAb and saporin on long term cultures of HSB-2 cells*

A series of experiments was undertaken to establish the effects of various concentrations of saporin and BsAb on HSB-2 cell proliferation and protein synthesis in cell culture measured daily over a 10 day period.

In the first set of experiments cultures of cells were continuously exposed to a fixed BsAb concentration of 0.1 $\mu g\,ml^{-1}$ in the presence of varying concentrations of saporin ranging from 0.000001 $\mu g\,ml^{-1}$ (0.33 pmol) to 0.1 $\mu g\,ml^{-1}$ (3.3 nmol) and daily cell counts and $^3$H-leucine incorporations determined over a 10 day period. Figure 6 shows the results obtained and demonstrates a very good

**Figure 5** Blockage of BsAb-mediated delivery of saporin to HSB-2 cells by a 10-fold excess of $F(ab'\gamma)_2$ of the anti-CD7 MoAb HB2. Leucine incorporation was measured in HSB-2 cells following a 48 h exposure to varying amounts of saporin together with $F(ab')_2$ (■——■) $IC_{50}$ 0.18 $\mu$mol, $F(ab')_2$ + HB2 × DB7-18 BsAb (□——□) $IC_{50}$ 73 nmol, or HB2 × DB7-18 BsAb (▲——▲) $IC_{50}$ 0.22 nmol.

**Figure 6** Viable cell counts **a**, and $^3$H-leucine incorporation expressed as decays per minute (d.p.m.), **b**, measured over a 10 day period in cultures of HSB-2 cells exposed continuously to 0.1 $\mu g\,ml^{-1}$ HB2 × DB7-18 BsAb together with various concentrations of saporin. No saporin (■——■) or saporin at 0.000001 $\mu g\,ml^{-1}$ (◇——◇), 0.00001 $\mu g\,ml^{-1}$ (○——○), 0.0001 $\mu g\,ml^{-1}$ (●——●), 0.001 $\mu g\,ml^{-1}$ (□——□), 0.01 $\mu g\,ml^{-1}$ (▽——▽) and 0.1 $\mu g\,ml^{-1}$ (▲——▲).

**Figure 4** Protein synthesis levels in HPB-ALL and HL60 cells following exposure to varying concentrations of saporin alone or in combination with BsAb. HL60; saporin only $IC_{50}$ 90 nmol (□——□) HL60; BsAb + Saporin $IC_{50}$ 90 nmol, (△——△) HPB-ALL; saporin alone $IC_{50}$ 51 nmol (◆——◆) and HPB-ALL; BsAb + saporin (▼——▼) $IC_{50}$ 22 nmol.

correlation between the viable cell count data (Figure 6a) and leucine incorporation data (Figure 6b). The dose response to saporin concentration in the presence of a fixed BsAb concentration of 0.1 $\mu g\,ml^{-1}$ is clearly obvious with 0.01 $\mu g\,ml^{-1}$ of saporin giving complete and total inhibition of both cell division and leucine incorporation. Microscopic examination at 10 days revealed that the vast majority of cells in these cultures were non-viable with very rare, apparently viable cells occasionally visible in the culture. At a saporin concentration of 0.001 $\mu g\,ml^{-1}$ cell division was slowed to approximately two thirds that obtained with untreated control HSB-2 cultures whilst leucine incorporation was reduced to less than half that seen in the control cultures. Saporin concentrations of 0.0001 $\mu g\,ml^{-1}$ (3.3 pmol) or less had little or no effect.

In similar experiments we continuously exposed cultures of HSB-2 cells to saporin at two fixed concentrations of 0.1 $\mu g\,ml^{-1}$ (3.3 nmol) (Figures 7a and b) or 0.01 $\mu g\,ml^{-1}$ (0.33 nmol) (Figures 7c and d) in the presence of various concentrations of BsAb ranging from 0.001 $\mu g\,ml^{-1}$ to 0.1 $\mu g\,ml^{-1}$. Viable cell counts and $^{3}H$-leucine incorporation was determined for each culture daily over the 10 day period of the experiment and the results presented as a percentage of control cultures. The dose response to BsAb concentration over the time course of the experiment is clearly obvious in cultures treated with 0.1 $\mu g\,ml^{-1}$ saporin (Figures 7a and b), there being an extremely good correlation between viable cell count and leucine incorporation over the range of BsAb concentrations used. Thus, BsAb used at 0.01 and 0.1 $\mu g\,ml^{-1}$ was effective at totally inhibiting HSB-2 cell division and protein synthesis at a saporin concentration of 0.1 $\mu g\,ml^{-1}$ whilst cells treated with 0.005 $\mu g\,ml^{-1}$ BsAb showed a shallower reduction and cell proliferation and protein synthesis began to increase once again by day 9 (Figures 7a and b). At a BsAb concentration of 0.001 $\mu g\,ml^{-1}$ viable cell count and protein synthesis levels dropped to a nadir by day 5 but began to recover thereafter achieving control levels by day 8.

At a saporin concentration of 0.01 $\mu g\,ml^{-1}$ (Figures 7c and d) the only effective BsAb concentration which completely inhibited cell proliferation and protein synthesis in the cultures was 0.1 $\mu g\,ml^{-1}$. BsAb concentrations of 0.005 $\mu g\,ml^{-1}$ and 0.01 $\mu g\,ml^{-1}$ both caused a transient depression in both viable cell count (Figure 7c) and protein synthesis levels (Figure 7d) which reached their nadir by day 5 showing approximately half the values seen in control cultures. However, by day 7 to 8 both viable cell count and protein synthesis levels began to increase reaching control levels by the end of the experiment.

*Multiple treatment studies*

In order to establish whether surviving HSB-2 cells previously treated with saporin and BsAb were more resistant to subsequent rounds of treatment we conducted the following experiments. HSB-2 cells surviving continuous exposure to 0.1 $\mu g\,ml^{-1}$ saporin + 0.01 $\mu g\,ml^{-1}$ BsAb from the experiment shown in Figures 7a and b began to divide after 10 days in culture. By day 17 there were sufficient cells ($5 \times 10^{5}$) to repeat a further round of treatment with the same levels of BsAb and saporin. Once HSB-2 cells surviving this second round of treatment, began dividing again, they were washed, put into fresh complete RPMI medium and grown to high density prior to investigation. These twice treated HSB-2 cells were designated HSB-2(G +). Prior to study HSB-2(G +) and untreated HSB-2 cells were analysed by flow cytometry for cell surface CD7 expression using the native anti-CD7 MoAb HB2. There were no observed differences in the level of cell surface expresson of CD7 between the native HSB-2 cells and HSB-2(G +) cells (data not shown).

HSB-2(G +) cells were exposed for 48 h to increasing concentrations of saporin (range $10^{-12}$ to $10^{-6}$ M) together with BsAb (0.1 $\mu g\,ml^{-1}$) and $^{3}H$-leucine incorporation evaluated. HSB-2(G +) cells were still found to be sensitive to BsAb/saporin treatment with an observed $IC_{50}$ of 0.38 nmol, this representing a 579-fold increase in toxicity above that seen with saporin alone ($IC_{50}$ 0.22 $\mu$mol) in this particular experiment (data not shown).

**Figure 7** Viable cell counts **a**, and **c** and $^{3}H$-leucine incorporation expressed as a percentage of control **b**, and **d**, measured over a 10 day period in HSB-2 cell cultures exposed to either 0.1 $\mu g\,ml^{-1}$ saporin **a** and **b**, or 0.01 $\mu g\,ml^{-1}$ saporin **c** and **d**, together with various concentrations of HB2 × DB7-18 BsAb BsAb at 0.001 $\mu g\,ml^{-1}$ (◇——◇), 0.005 $\mu g\,ml^{-1}$ (◆——◆), 0.01 $\mu g\,ml^{-1}$ (△——△) and 0.1 $\mu g\,ml^{-1}$ (▲——▲). Cultures were also exposed to saporin alone (■——■) and BsAb alone (□——□).

### Discussion

The present study has clearly shown that a $F(ab'\gamma)_2$ bispecific antibody (BsAb) with dual anti-CD7 and anti-saporin specificities can effectively and specifically deliver a cytotoxic dose of saporin to the $CD7^+$ T-ALL cell line HSB-2. The cytotoxic response following BsAb-mediated delivery of saporin to HSB-2 cells was dose dependent in terms of both BsAb and saporin concentrations. We were also able to demonstrate a very good correlation between BsAb/saporin-mediated inhibition of cell proliferation and inhibition of protein synthesis in 10 day cell cultures and moreover observe that cell death was actually occurring in these cultures.

CD7 has been successfully used as the target molecule on a variety of fresh and established human leukaemia cell lines for targeting intact ricin (Strong *et al.*, 1985), ricin A chain (Myers *et al.*, 1984) and single chain ribosome inactivating proteins (*rip*) (Ramakrishnan & Houston, 1984). An immunotoxin constructed with the anti-CD7 MoAb 3A1 and the *rip* pokeweed antiviral protein (PAP) was highly cytotoxic for the T-ALL cell line HSB-2 with an observed $IC_{50}$ of 0.11 nmol (Ramakrishnan & Houston, 1984). This $IC_{50}$ value is very close to that obtained for BsAb-mediated delivery of saporin via the CD7 molecule also on HSB-2 cells ($IC_{50}$ 0.23 nmol) observed in the present study and suggests that BsAb can deliver toxin with a similar efficiency as IT. This is an interesting observation as BsAb delivers rip to the cell surface univalently without cross linking adjacent CD7 molecules on the cell surface. This is in contrast to IT's constructed with intact immunoglobulin molecules which are bivalent and therefore do cross link adjacent target molecules on the cell surface. Thus, one might expect IT's to deliver the toxin/rip more effectively by virtue of increased binding affinity at the cell surface through two target molecules and perhaps more importantly due to the increased rate of modulation and subsequent internalisation that occurs when adjacent target molecules are cross linked at the cell surface. Unfortunately, there are to the best of our knowledge no published reports of anti-CD7 IT's constructed with saporin and therefore valid statements about BsAb *vs* IT-mediated delivery of saporin cannot really be made until these have been compared directly on the same cell line. We are planning experiments to address this issue in the near future.

Saporin is a single chain ribosome inactivating protein (Stirpe *et al.*, 1983) and unlike intact ricin does not have any intrinsic means of entering the cell and therefore becomes cytotoxic only at relatively high concentrations (in the present study the $IC_{50}$ of saporin for HSB-2 cells was 0.1 $\mu$mol). The toxicity that is seen at relatively high concentrations of saporin is probably due to the entry of small amounts of saporin into the cell by fluid pinocytosis. BsAb reduced the $IC_{50}$ for HSB-2 to 0.23 nmol, a 435-fold increase in toxicity over saporin alone. The specificity of saporin delivery by BsAb was unequivocally demonstrated by two different methods. In the first BsAb + saporin was shown to have no cytotoxic effect on the $CD7^-$ cell line HL60 and had only a small effect on the weak $CD7^+$ positive cell line HPB-ALL. The two and a half-fold decrease in $IC_{50}$ that BsAb gave for HPB-ALL almost certainly represents the delivery of only a small amount of saporin to the target ribosomes in the cell interior due to the very low or non-existant density of target antigen expression by the majority of HPB-ALL cells. As one might expect, this clearly demonstrates the importance of the level of cell surface target antigen expression that has been commented on previously by other workers (Laurent *et al.*, 1986).

The observation that HSB-2(G +) cells that had been put through two cycles of BsAb and saporin treatment were equally as sensitive as previously untreated HSB-2 cells, to subsequent rounds of BsAb/saporin treatment provides an important clue as to how these cells escaped destruction in the first instance. There were no observed differences between HSB-2(G +) and HSB-2 cells for cell surface expression of CD7 showing that a subpopulation of variant target antigen negative cells had not been selected for by BsAb/saporin treatment. This is unlike the findings of Glennie *et al.* (1988) where target guinea pig $L_2C$ tumour cells emerging following BsAb/saporin treatment *in vivo* were target antigen negative. Similarly, a transferrin-Ricin A chain resistant clone of the T-cell line CEM had reduced levels of expression of the target transferrin receptor molecule (CD71) (Raso & Basala, 1984) though it has not always been possible to correlate sensitivity to an immunotoxin and the level of expression of the target antigen by the target cell (Goldmacher *et al.*, 1987). The retained sensitivity of HSB-2(G +) cells to BsAb/saporin also excludes the possibility that we had selected for a subpopulation of HSB-2 cells with an inherent defect in endocytosis transport of BsAb-saporin complexes across the membrane (Goldmacher *et al.*, 1987), or with endosome-cytosol translocation of toxin to the target ribosome. We feel that the most likely explanation is quite simply that a small proportion of the original HSB-2 cells at the time of BsAb/saporin treatment were negative or weakly positive for CD7 expression and therefore escaped lethal intoxication at this time. Following their subsequent outgrowth some weeks later, by which time the majority of the BsAb/saporin had been consumed or had degraded in culture, these cells then upregulated their CD7 expression and were therefore accessible to saporin delivery with the BsAb when so treated on a future occasion. This likely possibility really underlines the potential shortcomings of toxin delivery *via* a single target molecule and argues strongly for using multiple target molecules for toxin delivery, firstly to overcome the heterogeneity of target molecule expression within a tumour cell population and secondly to deliver greater amounts of toxin to those tumour cells that are positive for all target molecules.

IT's have been used for the *ex vivo* purging of residual leukaemic blasts from bone marrow harvested from acute leukaemia patients in remission prior to autografting (Strong *et al.*, 1985). Autografting is a significantly less toxic procedure than allografting but is also less successful with the majority of patients relapsing post-transplant (Kersey *et al.*, 1987). Indications are that the graft *vs* host disease that often accompanies allogeneic transplantation provides an effective anti-leukaemia response (graft *vs* leukaemia) that serves to eliminate residual leukaemic blasts within the patient (Butturini *et al.*, 1987). It is this particular effect, absent in autologous transplantation, that might be partially, if not wholly responsible for the lower success rate of autografting. In view of the high toxicity of the allografting procedure and the limited availability of matched donors, methods for improving the success rate in autografting would represent a major step forward in the treatment of relapsed disease. It is not clear whether the relapses that occur following autografting do so from residual leukaemic stem cells remaining in the patient or in the reinfused marrow inoculum. In practice both may contribute and future objectives should aim to reduce residual tumour cell numbers to the absolute minimum achievable. Thus, *in vivo* administration of IT's during conditioning *prior* to transplant, for the elimination of residual disease in the patient combined with *ex vivo* treatment of the patients own harvested marrow prior to reinfusion may lead to some improvement. There is thus a strong case for developing IT type therapies for *in vivo* use and recently small clinical studies have been undertaken in leukaemia (Laurent *et al.*, 1988; Byers *et al.*, 1990) and other malignant tumour types (Spitler, 1988).

The clinical utility of BsAb's for targeting toxins to unwanted neoplastic cells *in vivo* yet remains to be seriously explored. Glennie *et al.* (1988) have undertaken *in vivo* immunotherapy studies in guinea pigs bearing the guinea pig B-lineage acute lymphoblastic leukaemia tumour $L_2C$, targeting saporin against an idiotypic determinant on the tumour cell surface with a BsAb. In these studies there was a significant prolongation of survival in animals receiving saporin and BsAb, the extent of which was determined by the route of administration and molar ratio of BsAb to saporin employed. It is essential that the behaviour and performance of BsAb's for delivering toxins to human leukaemia cells is thoroughly investigated *in vivo* in an animal xenograft model prior to any attempt to utilise them clinically. To this end we are actively developing a model of human acute leukaemia in severe combined immunodeficient (*scid*) mice (Kamel-Reid *et al.*, 1989) with the objective of commencing immunotherapy trials with BsAb's in these animals in the very near future.

We would like to thank Dr Martin Glennie and Ms Alison Tutt (Tenovus Laboratory, Southampton) for constructing the HB2 x BD7-18 BsAb. This work was supported in part by Leukaemia Busters and Lederle Cyanamid, UK. D.J.F. and S.U.F. are Cancer Research Campaign Research Fellows.

### References

BLAKEY, D.C., SKILLETER, D.N., PRICE, R.J. & 4 others (1988). Comparison of the pharmacokinetics and hepatotoxic effects of saporin and ricin A-chain immunotoxins on murine liver parenchymal cells. *Cancer Res.*, **48**, 7072.

BLYTHMAN, H.E., BORD, A., BUISSON, I., THURNEYSSEN, O., RICHER, G. & JANSEN, F.K. (1986). The nude mouse for the study of immunotoxins. In *Human Tumour Xenografts for Anticancer Drug Development*, Pinedo, H.M., Peckham, M.J. & Winogrid, B. (eds), Springer Verlag.

BREGNI, M., SIENA, S., FORMOSA & 6 others (1989). B-cell restricted saporin immunotoxins. Activity against B-cell lines and chronic lymphocytic leukaemia cells. *Blood*, **73**, 753.

BUTTURINI, A., BORTIN, M.M. & GALE, R.P. (1987). Graft *versus* leukaemia following bone marrow transplantation. *Bone Marrow Transplant.*, **2**, 233.

BYER, V.S., DUERST, R., CARROLL, S. & 4 others (1990). Use of an anti-CD7-Ricin A chain immunotoxin in the treatment of T-cell acute lymphocytic leukaemia. (Abstract) Second International Symposium on Immunotoxins. Lake Buena Vista, Florida, June 1990.

CASELLAS, P., BOURRIE, B.J., GROS, P. & JANSEN, F.K. (1984). Kinetics of cytotoxicity induced by immunotoxins. Enhancement by lysomotropic amines and carboxylic ionophores. *J. Biol. Chem.*, **259**, 9359.

CHAMPLIN, R. & GALE, R.P. (1989). Acute lymphoblastic leukaemia: recent advances in biology and therapy. *Blood*, **73**, 2051.

CHESSELLS, J.M. (1987). The acute lymphoblastic leukaemias. In *Leukaemia*. Whittaker & Delamore (eds), Blackwell Scientific Publication, pp. 331.

FITZGERALD, D.J., WILLINGHAM, M. & PASTAN, I. (1986). Antitumour effect of an immunotoxin made with *Pseudomonas* exotoxin in a nude mouse model of human ovarian cancer. *Proc. Natl Acad. Sci. USA*, **83**, 6627.

GHETIE, M.A., MAY, R.D., TILL, M. & 10 others (1988). Evaluation of ricin A chain-containing immunotoxins directed against CD19 and CD22 antigens on normal and malignant tumour B-cells as potential reagents for *in vivo* therapy. *Cancer Res.*, **48**, 2610.

GLENNIE, M.J., MCBRIDE, H.M., WORTH, A.T. & STEVENSON, G.T. (1987). Preparation and performance of bispecific $F(ab')_2$ antibody containing thioether-linked Fab' fragments. *J. Immunol.*, **139**, 2367.

GLENNIE, M.J., BRENNAND, D.M., BRYDEN, F. & 4 others (1988). Bispecific $F(AB')_2$ antibody for the delivery of saporin in the treatment of lymphoma. *J. Immunol.*, **141**, 3662.

GOLDMACHER, V.S., ANDERSON, J., SCHULZ, M.L., BLUTLER, W.A. & LAMBERT, J.M. (1987). Somatic cell mutants resistant to ricin, diphtheria toxin and to immunotoxins. *J. Biol. Chem.*, **262**, 3205.

HERTLER, A.A. & FRANKEL, A.E. (1989). Immunotoxins:. a clinical review of their use in the treatment of malignancies. *J. Clin. Oncol.*, **7**, 1932.

KAMEL-REID, S., LETARTE, M. SIRARD, C. & 6 others (1989). A model of human acute lymphoblastic leukaemia in immune deficient scid mice. *Science*, **246**, 1597.

KERSEY, J.H., WEISDORF, D., NESBIT, M.E. & 9 others (1987). Comparison of autologous and allogeneic bone marrow transplantation for treatment of high-risk refractory acute lymphoblastic leukaemia. *New Engl. J. Med.*, **317**, 461.

LAURENT, G., KUHLEIN, E., CASELLAS, P. & 6 others (1986). Determination of sensitivity of fresh leukaemic cells to immunotoxins. *Cancer Res.*, **46**, 2289.

LAURENT, G., FRANKEL, A.E., HERTLER, A.A., SCHLOSSMAN, P.M., CASELLAS, P. & JANSEN, F.K. (1988). Treatment of leukaemia patients with T101 ricin A chain immunotoxins. In *Immunotoxins*. Frankel, A.E. (ed.) Kluwar Academic Publishers: Boston, pp. 483.

MYERS, C.D., THORPE, P.E., ROSS, W.C.J. & 4 others (1984). An immunotoxin with therapeutic potential in T-cell leukaemia TW1 Ricin A. *Blood*, **63**, 1178.

PRESS, O.W., HANSEN, J.A., FARR, A. & MARTIN, P.J. (1988). Endocytosis and degradation of murine anti-human CD3 monoclonal antibodies by normal and malignant T-lymphocytes. *Cancer Res.*, **48**, 2249.

RAMAKRISHNAN, S. & HOUSTON, L.L. (1984). Inhibition of human acute lymphoblastic leukaemia cells by immunotoxins. Potentiation by chloroquine. *Science*, **223**, 58.

RASO, V. & BASALA, M. (1984). A highly cytotoxic human transferrin-ricin A chain conjugate used to select receptor modified cells. *J. Biol. Chem.*, **259**, 1143.

REPORT FROM THE WORKING PARTY ON LEUKAEMIA. EUROPEAN GROUP FOR BONE MARROW TRANSPLANTATION (1988). Allogeneic bone marrow transplantation for leukaemia in Europe. *Lancet*, **ii**, 1379.

SCOTT, C.J. Jr, LAMBERT, J.M., GOLDMACHER, V.S., BENACERRAF, B. (1987). The pharmacokinetic and toxicity of murine monoclonal antibodies and of gelonin conjugates of their antibodies. *Int. J. Immunopharmacol.*, **9**, 211.

SPITLER, L.E. (1988). Clinical Studies: solid tumours. In *Immunotoxins*. Frankel, A.E. (ed.) Kluwer Academic Publishers: Boston, p. 493.

STIRPE, F., GASPERI-CAMPANI, G., BARBIERI, L., FALASCA, A., ABBONDANZA, A. & STEVENS, W.A. (1983). Ribosome inactivating proteins from the seeds of *Saponaria officinalis L.* (soapwort), of *Agrostemma githago L.* (corncockle) and of *Asparagus officinalis* (asparagus) and from latex of *Hura crepitans L.* (sandbox tree). *Biochem. J.*, **216**, 617.

STRONG, R.C., UCKUN, F., YOULE, R.J., KERSEY, J.H. & VALLERA, D.A. (1985). Use of multiple T-cell directed intact ricin immunotoxins for autologous bone marrow transplantation. *Blood*, **66**, 627.

THORPE, P., BROWN, A., BREAMER, J., FOXWELL, B. & STIRPE, F. (1985). An immunotoxin composed of monoclonal anti-Thy 1.1 antibody and a ribosome inactivating protein for *Saponaria officinalis*. Potent anti tumour effect *in vitro* and *in vivo*. *J. Natl Cancer Inst.*, **75**, 151.

THORPE, P.E., WALLACE, P.M., KNOWLES, P.P. & 5 others (1988). Improved antitumour effects of immunotoxins prepared with deglycosylated ricin A-chain and hindered disulphide linkages. *Cancer Res.*, **48**, 6396.

WEBB, K.S., WARE, J.L., PARKS, S.F., WALTHER, P.J. & PAULSON, D.F. (1985). Evidence for a novel hybrid immunotoxin recognising ricin A chain by one antigen combining site and a prostate-restricted antigen by the remaining antigen-combining site: potential for immunotherapy. *Cancer Treat. Reports*, **69**, 663.


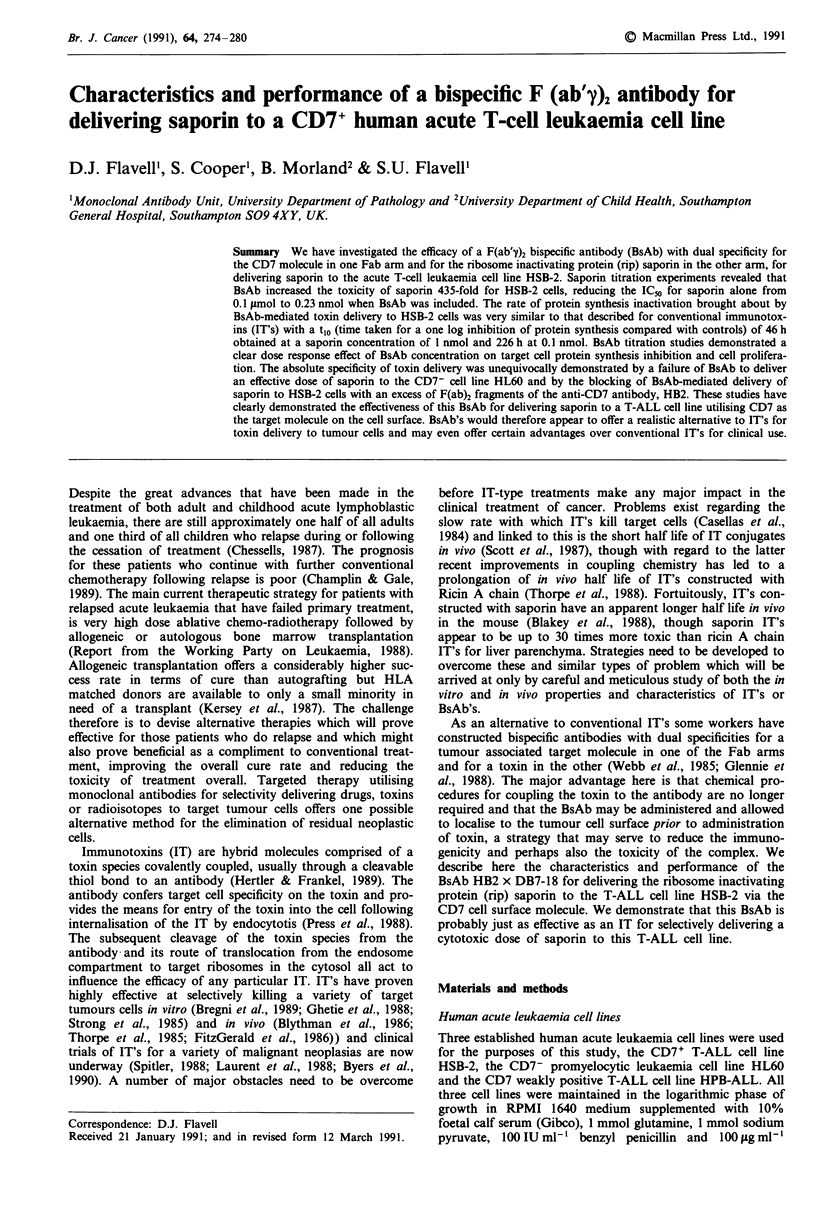

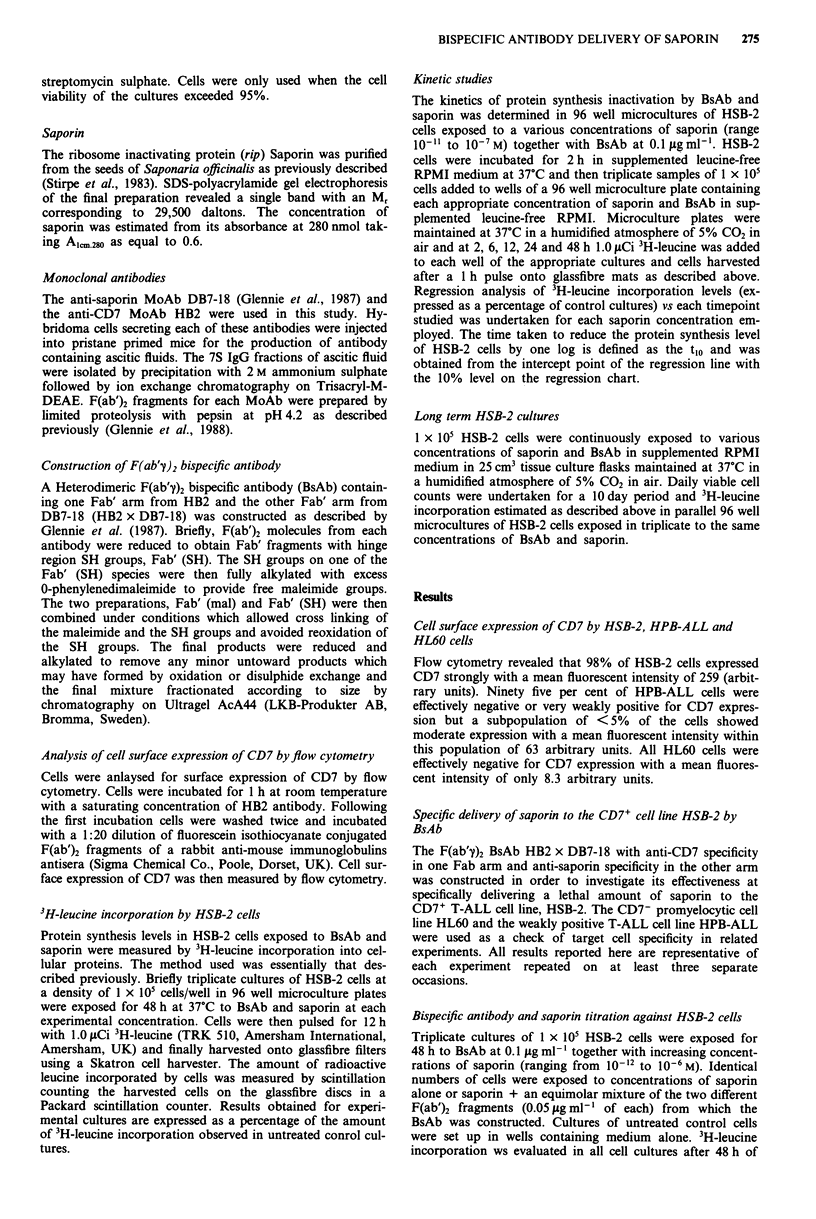

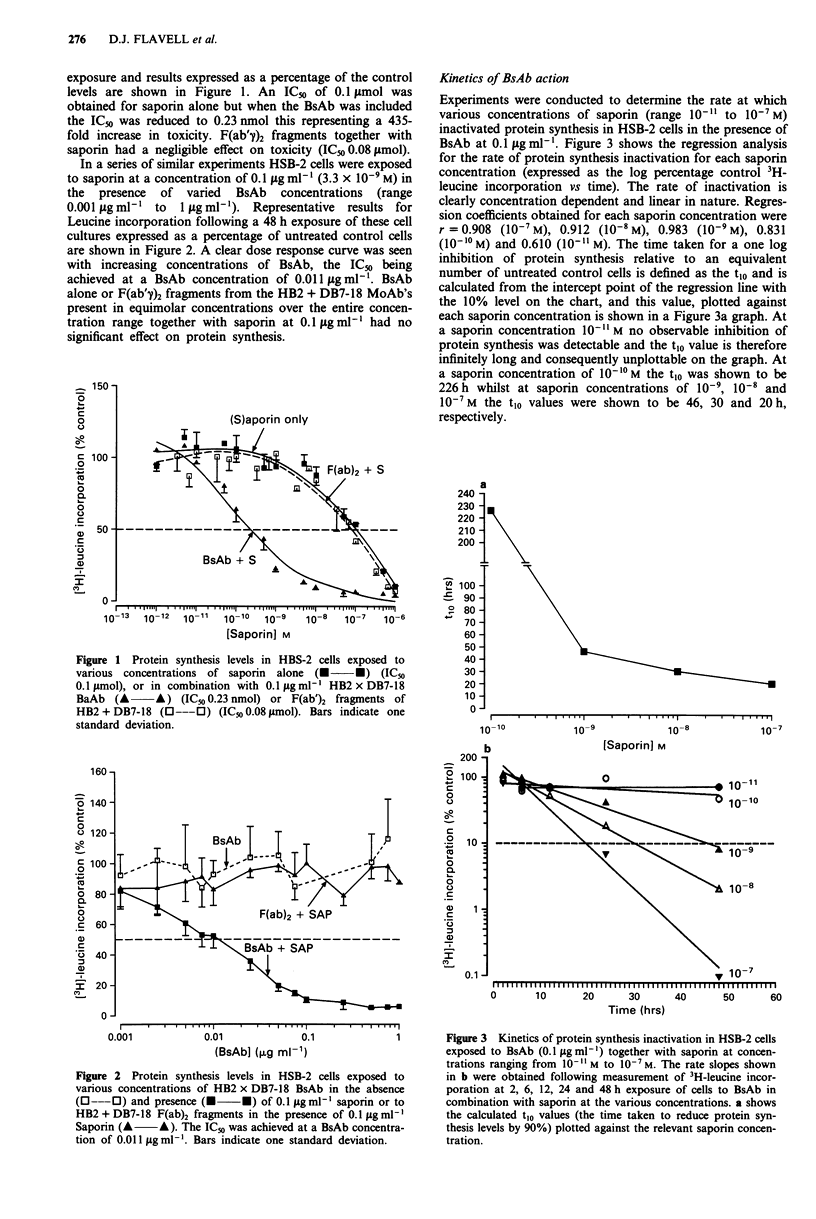

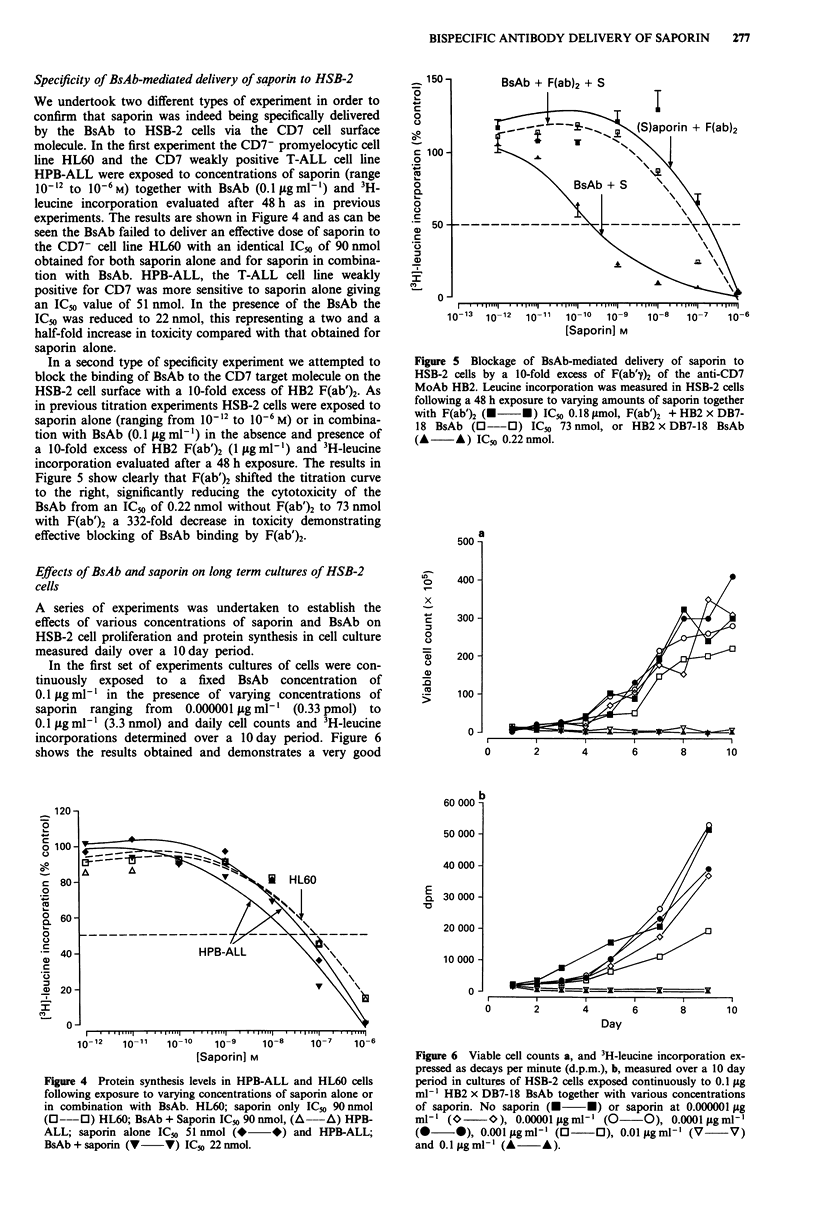

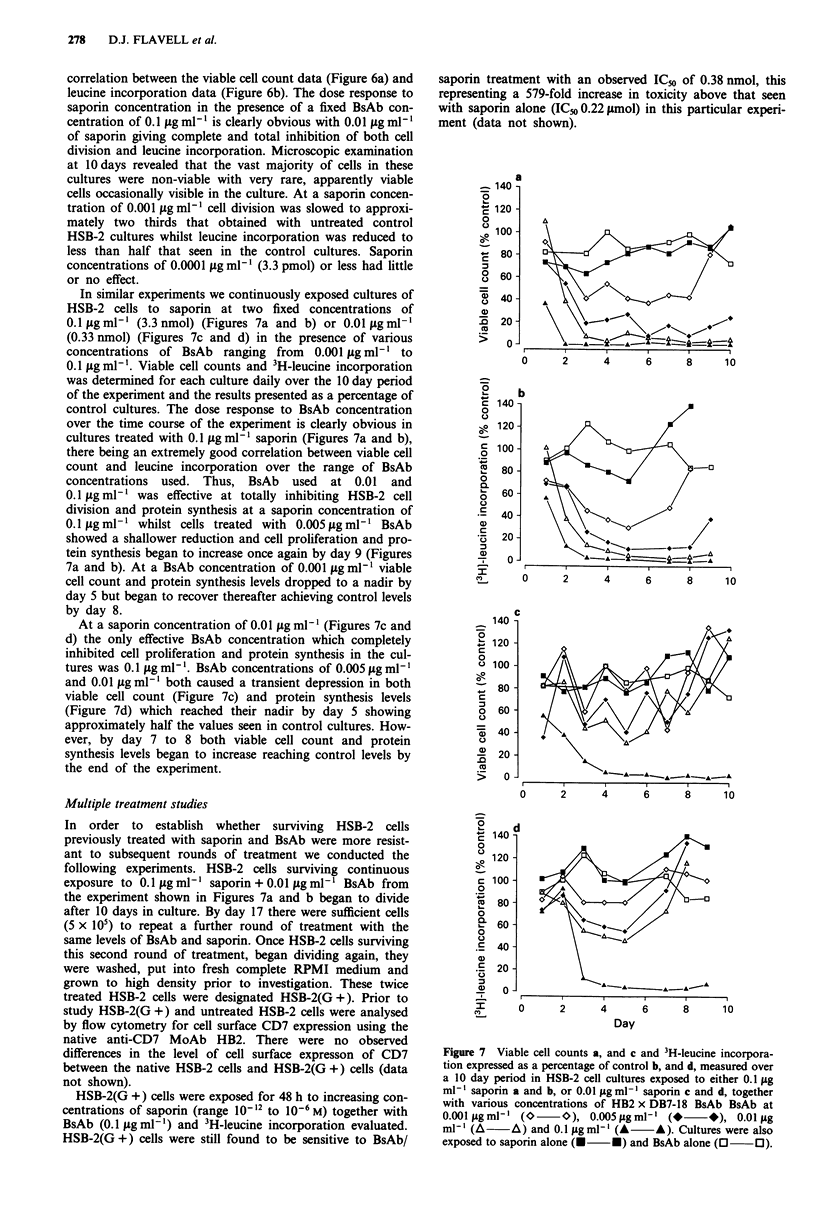

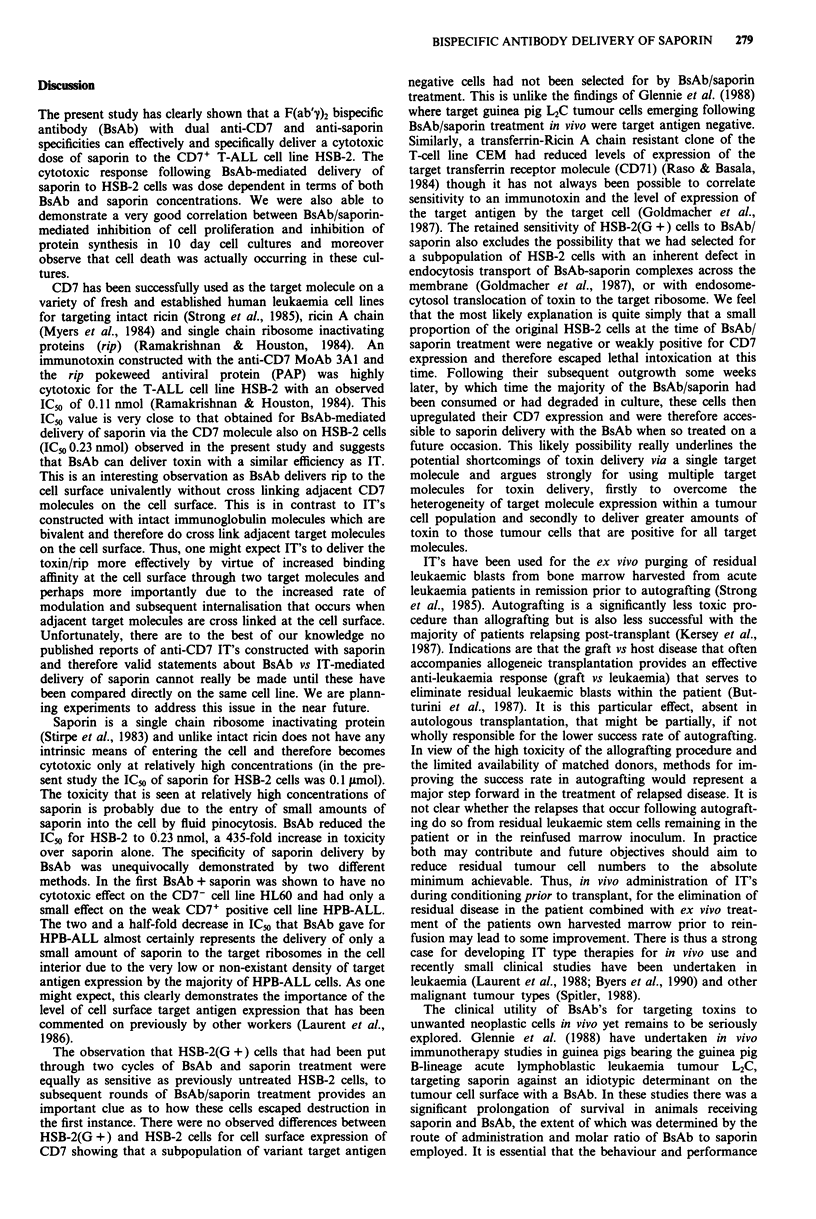

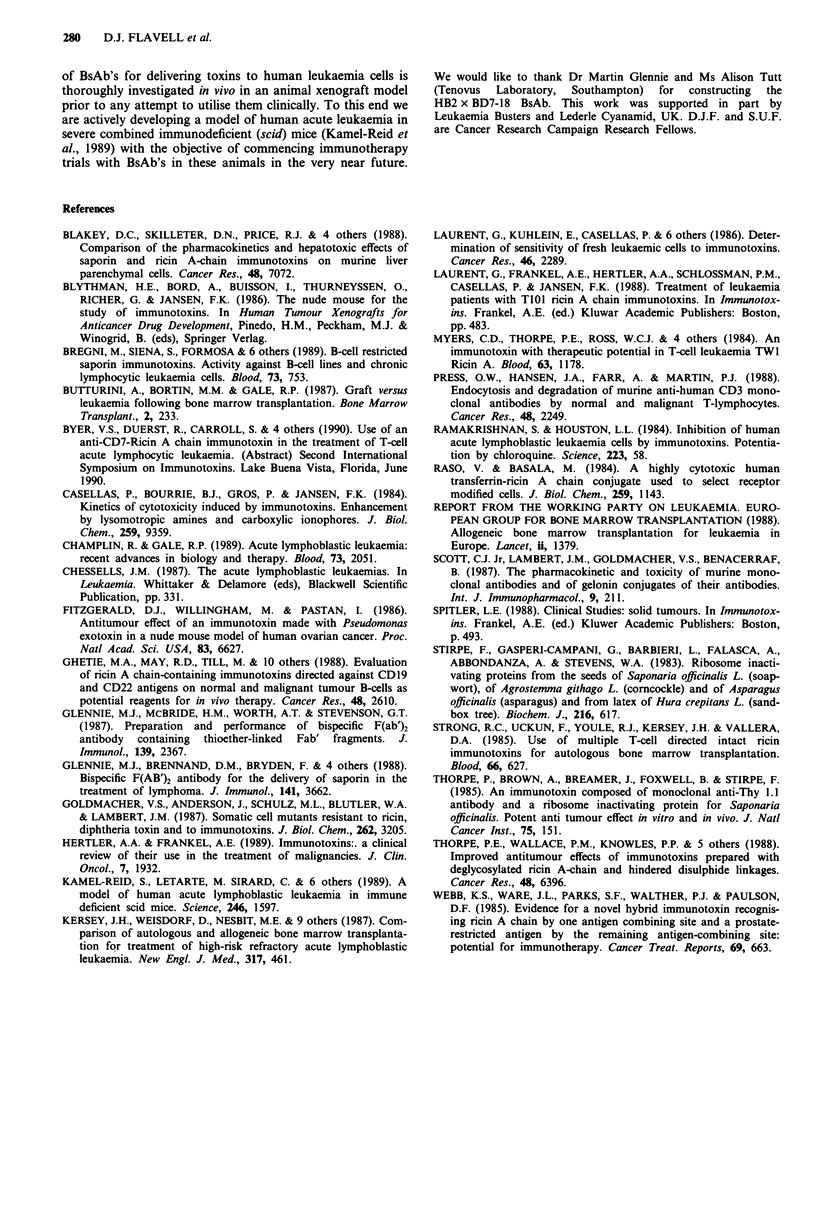


## References

[OCR_01149] Blakey D. C., Skilleter D. N., Price R. J., Watson G. J., Hart L. I., Newell D. R., Thorpe P. E. (1988). Comparison of the pharmacokinetics and hepatotoxic effects of saporin and ricin A-chain immunotoxins on murine liver parenchymal cells.. Cancer Res.

[OCR_01160] Bregni M., Siena S., Formosa A., Lappi D. A., Martineau D., Malavasi F., Dorken B., Bonadonna G., Gianni A. M. (1989). B-cell restricted saporin immunotoxins: activity against B-cell lines and chronic lymphocytic leukemia cells.. Blood.

[OCR_01165] Butturini A., Bortin M. M., Gale R. P. (1987). Graft-versus-leukemia following bone marrow transplantation.. Bone Marrow Transplant.

[OCR_01177] Casellas P., Bourrie B. J., Gros P., Jansen F. K. (1984). Kinetics of cytotoxicity induced by immunotoxins. Enhancement by lysosomotropic amines and carboxylic ionophores.. J Biol Chem.

[OCR_01183] Champlin R., Gale R. P. (1989). Acute lymphoblastic leukemia: recent advances in biology and therapy.. Blood.

[OCR_01192] FitzGerald D. J., Willingham M. C., Pastan I. (1986). Antitumor effects of an immunotoxin made with Pseudomonas exotoxin in a nude mouse model of human ovarian cancer.. Proc Natl Acad Sci U S A.

[OCR_01198] Ghetie M. A., May R. D., Till M., Uhr J. W., Ghetie V., Knowles P. P., Relf M., Brown A., Wallace P. M., Janossy G. (1988). Evaluation of ricin A chain-containing immunotoxins directed against CD19 and CD22 antigens on normal and malignant human B-cells as potential reagents for in vivo therapy.. Cancer Res.

[OCR_01210] Glennie M. J., Brennand D. M., Bryden F., McBride H. M., Stirpe F., Worth A. T., Stevenson G. T. (1988). Bispecific F(ab' gamma)2 antibody for the delivery of saporin in the treatment of lymphoma.. J Immunol.

[OCR_01204] Glennie M. J., McBride H. M., Worth A. T., Stevenson G. T. (1987). Preparation and performance of bispecific F(ab' gamma)2 antibody containing thioether-linked Fab' gamma fragments.. J Immunol.

[OCR_01215] Goldmacher V. S., Anderson J., Schulz M. L., Blättler W. A., Lambert J. M. (1987). Somatic cell mutants resistant to ricin, diphtheria toxin, and to immunotoxins.. J Biol Chem.

[OCR_01219] Hertler A. A., Frankel A. E. (1989). Immunotoxins: a clinical review of their use in the treatment of malignancies.. J Clin Oncol.

[OCR_01224] Kamel-Reid S., Letarte M., Sirard C., Doedens M., Grunberger T., Fulop G., Freedman M. H., Phillips R. A., Dick J. E. (1989). A model of human acute lymphoblastic leukemia in immune-deficient SCID mice.. Science.

[OCR_01231] Kersey J. H., Weisdorf D., Nesbit M. E., LeBien T. W., Woods W. G., McGlave P. B., Kim T., Vallera D. A., Goldman A. I., Bostrom B. (1987). Comparison of autologous and allogeneic bone marrow transplantation for treatment of high-risk refractory acute lymphoblastic leukemia.. N Engl J Med.

[OCR_01242] Laurent G., Frankel A. E., Hertler A. A., Schlossman D. M., Casellas P., Jansen F. K. (1988). Treatment of leukemia patients with T101 ricin A chain immunotoxins.. Cancer Treat Res.

[OCR_01237] Laurent G., Kuhlein E., Casellas P., Canat X., Carayon P., Poncelet P., Correll S., Rigal F., Jansen F. K. (1986). Determination of sensitivity of fresh leukemia cells to immunotoxins.. Cancer Res.

[OCR_01247] Myers C. D., Thorpe P. E., Ross W. C., Cumber A. J., Katz F. E., Tax W., Greaves M. F. (1984). An immunotoxin with therapeutic potential in T cell leukemia: WT1-ricin A.. Blood.

[OCR_01252] Press O. W., Hansen J. A., Farr A., Martin P. J. (1988). Endocytosis and degradation of murine anti-human CD3 monoclonal antibodies by normal and malignant T-lymphocytes.. Cancer Res.

[OCR_01258] Ramakrishnan S., Houston L. L. (1984). Inhibition of human acute lymphoblastic leukemia cells by immunotoxins: potentiation by chloroquine.. Science.

[OCR_01263] Raso V., Basala M. (1984). A highly cytotoxic human transferrin-ricin A chain conjugate used to select receptor-modified cells.. J Biol Chem.

[OCR_01274] Scott C. F., Lambert J. M., Goldmacher V. S., Blatter W. A., Sobel R., Schlossman S. F., Benacerraf B. (1987). The pharmacokinetics and toxicity of murine monoclonal antibodies and of gelonin conjugates of these antibodies.. Int J Immunopharmacol.

[OCR_01285] Stirpe F., Gasperi-Campani A., Barbieri L., Falasca A., Abbondanza A., Stevens W. A. (1983). Ribosome-inactivating proteins from the seeds of Saponaria officinalis L. (soapwort), of Agrostemma githago L. (corn cockle) and of Asparagus officinalis L. (asparagus), and from the latex of Hura crepitans L. (sandbox tree).. Biochem J.

[OCR_01293] Stong R. C., Uckun F., Youle R. J., Kersey J. H., Vallera D. A. (1985). Use of multiple T cell-directed intact ricin immunotoxins for autologous bone marrow transplantation.. Blood.

[OCR_01299] Thorpe P. E., Brown A. N., Bremner J. A., Foxwell B. M., Stirpe F. (1985). An immunotoxin composed of monoclonal anti-Thy 1.1 antibody and a ribosome-inactivating protein from Saponaria officinalis: potent antitumor effects in vitro and in vivo.. J Natl Cancer Inst.

[OCR_01306] Thorpe P. E., Wallace P. M., Knowles P. P., Relf M. G., Brown A. N., Watson G. J., Blakey D. C., Newell D. R. (1988). Improved antitumor effects of immunotoxins prepared with deglycosylated ricin A-chain and hindered disulfide linkages.. Cancer Res.

[OCR_01312] Webb K. S., Ware J. L., Parks S. F., Walther P. J., Paulson D. F. (1985). Evidence for a novel hybrid immunotoxin recognizing ricin A-chain by one antigen-combining site and a prostate-restricted antigen by the remaining antigen-combining site: potential for immunotherapy.. Cancer Treat Rep.

